# Supplementary material for: Epsilon-Near-Zero Grids for On-chip Quantum Networks
Source: Sci Rep. 2019 Apr 15;9:6053. doi: 10.1038/s41598-019-42477-3 (PMC6465304; doi:10.1038/s41598-019-42477-3)
Supplement: Supplementary file 1 — Suplemmentary material [file 41598_2019_42477_MOESM1_ESM.pdf]

# Epsilon-Near-Zero Grids for On-chip Quantum Networks

Larissa Vertchenko , Nika Akopian, and Andrei V. Lavrinenko

Department of Photonics Engineering, Technical University of Denmark, Ørsteds Plads 343, DK-2800 Kongens Lyngby, Denmark

January 31, 2019

Supplemental Material

Corresponding author: lariv@fotonik.dtu.dk

# Supercoupling theory

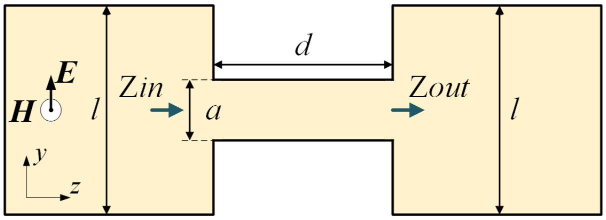


Figure 1: **ENZ waveguide**: Illustration of the impedance difference at the ports of the channel .

Considering the setup shown in Fig.1, where an incoming electric field polarized in the y direction, $E_y$, propagates through a waveguide with the variable cross section. The transversal wave impedance is defined as $Z = E_y/H_z$, where $H_z$ is the magnetic field. The transversal impedance of the waveguide before the channel is proportional to [1]

$$Z'_{in} \approx \frac{a}{l} Z_{in}, \tag{1}$$

and similarly the impedance after the channel is:

$$Z'_{out} \approx \frac{a}{l} Z_{out}, \tag{2}$$

where $Z_{in}$ and $Z_{out}$ are the impedances at the beginning and end of the channel, respectively, $a$ is the width of the ENZ channel and $l$ is the width of the waveguide before and after the channel. For the whole system the impedance difference between both ends of the channel is given by

$$\frac{Z_{out} - Z_{in}}{\eta_0} \approx i\mu k_0 d, \tag{3}$$

where $\eta_0 = \sqrt{\frac{\mu_0}{\epsilon_0}}$ is the free space impedance, $\mu$ is the relative permeability of the ENZ material, $k_0$ is the free-space wave number and $d$ the length ENZ channel. Substituting $Z_{in}$ and $Z_{out}$ in equation 3 by $Z'_{in}$ and $Z'_{out}$ results in

$$Z'_{out} - Z'_{in} \approx \frac{i\mu k_0 \eta_0 d a}{l}, \tag{4}$$

In order for the difference of the impedances between the input and output waveguides to be small at least one of the dimensions of the channel, either $d$ or $a$, has to be reduced.

## Optimization

The system was modeled by the finite element method, using the commercially available software COMSOL [2]. For a bounded ENZ medium with narrow channels the size of the air cavity was subjected to optimization by looking at the electric and magnetic field for different radius close to the resonance condition [3]

$$1 = \frac{c2\pi r}{A\omega_p}\frac{J_0'(2\pi r/\lambda)}{J_0(2\pi r/\lambda)}, \tag{5}$$

where $A$ is the area of the square, $c$ is the speed of light, $\omega_p$ the plasma frequency, $r$ the radius of the circle, $J_0$ the cylindrical Bessel function of the first kind and zero order and $J_0'$ the derivative of $J_0$ with respect to the argument. Fig. 2 shows the plots of the normalized electric and magnetic fields for a network of 5x5 and 15x15 cavities with different radius exhibiting the best result for $r = 310\ nm$.

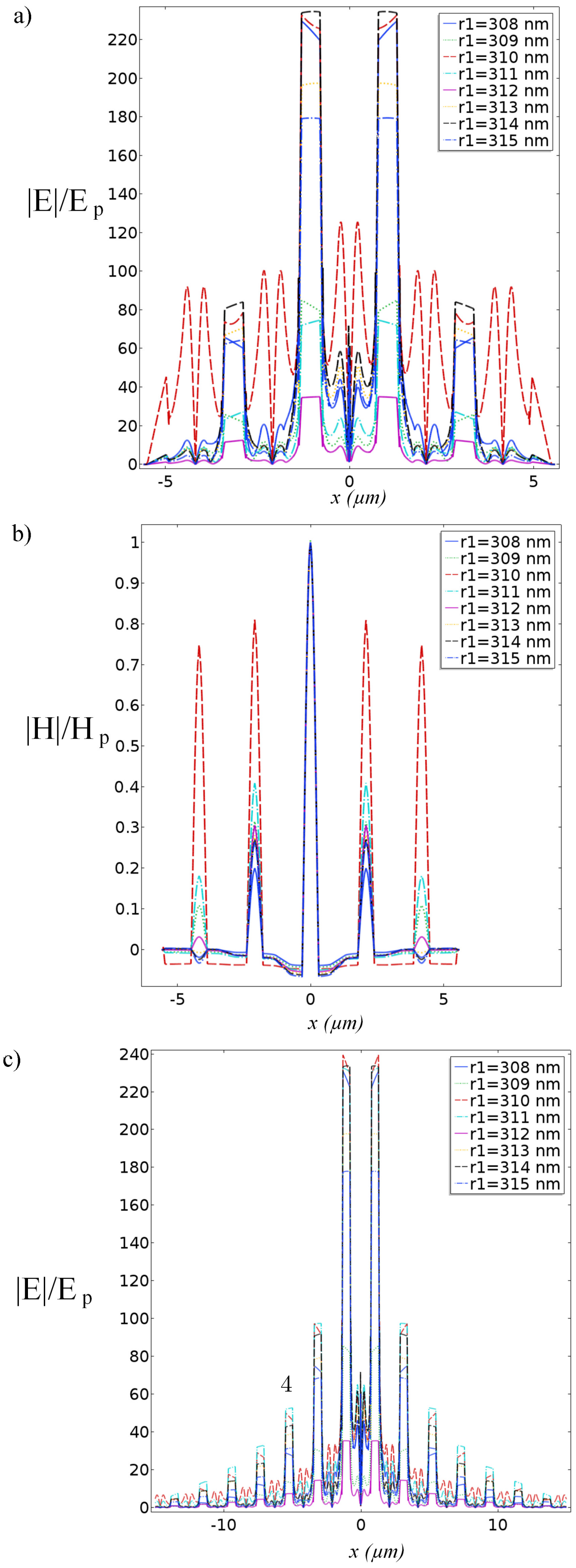
a)
|E|/E p
x (μm)
r1=308 nm
r1=309 nm
r1=310 nm
r1=311 nm
r1=312 nm
r1=313 nm
r1=314 nm
r1=315 nm
b)
|H|/H p
x (μm)
r1=308 nm
r1=309 nm
r1=310 nm
r1=311 nm
r1=312 nm
r1=313 nm
r1=314 nm
r1=315 nm
c)
|E|/E p
x (μm)
r1=308 nm
r1=309 nm
r1=310 nm
r1=311 nm
r1=312 nm
r1=313 nm
r1=314 nm
r1=315 nm

## Dispersion Model

In order to calculate the Lamb shift and decay rate due to coupling of two identical emitters we have used the dispersive Drude model of the form

$$\varepsilon(\omega) = 1 - \omega_p^2/\omega(\omega + i\gamma), \tag{6}$$

where $\omega_p$ is the plasma frequency corresponding to the wavelength of 780 nm and $\gamma$ characterizes the loss given by $\gamma = 0.001\omega_p$.

## Temporal coherence

The ENZ medium is represented by a Fermi gas with the dispersion relation given by [4]

$$\omega^2 = \omega_p^2 + \frac{3}{5}v_f^2 k^2 \tag{7}$$

where $\omega_p$ is the plasma frequency, $v_f$ is the Fermi velocity and $k$ is the wave number. Due to the imaginary part of the permittivity, the frequency turns into a complex value $\omega = \omega' + i\omega''$, with

$$\omega' = \omega_p \left(1 - \frac{1}{(2\omega\tau)^2}\right)^{1/2} \tag{8}$$

and

$$\omega'' = \frac{-1}{2\tau}, \tag{9}$$

where $\tau$ is the relaxation time of the plasmons, which can be estimated by Heisenberg's uncertainty principle

$$\Delta E \Delta\tau \geq \frac{\hbar}{2}, \tag{10}$$

where $\hbar$ is the reduced Planck constant and $\Delta E$ is the uncertainty on the energy. Using the loss function given by

$$loss_f = \frac{\epsilon''}{|\epsilon''|^2 + |\epsilon'|^2}, \tag{11}$$

where the complex permittivity is $\epsilon = \epsilon' + i\epsilon''$, we can estimate the frequency width, $\Delta f$, by the full width half maximum, and consequently $\Delta E$.

Using the well known equations for the autocorrelation function and the degree of temporal coherence [5] given by

$$G(\tau) = \int E^*(t)E(t+\tau)dt \tag{12}$$

and

$$g(\tau) = \frac{G(\tau)}{G(0)}, \tag{13}$$

respectively, where $E(T)$ is the electric field at a time $t$, we are able to find the coherence time from

$$\tau_c = \int |g(\tau)|^2 d\tau. \tag{14}$$

For the SiC at its ENZ point (10.32 $\mu m$), a coherence time of $1.061 \times 10^{-12} s$ was found. Considering that the mode propagates with a phase velocity equals to $\omega/k$ we find a coherence length value of 1.4 $mm$.

## SiC and TiN permittivity curves

The permittivity of a thin film of 117 $nm$ of TiN was measured using ellipsometry [6]. We emphasize that TiN properties highly depend on fabrication procedures. The data of SiC permittivity was taken from Ref [7].

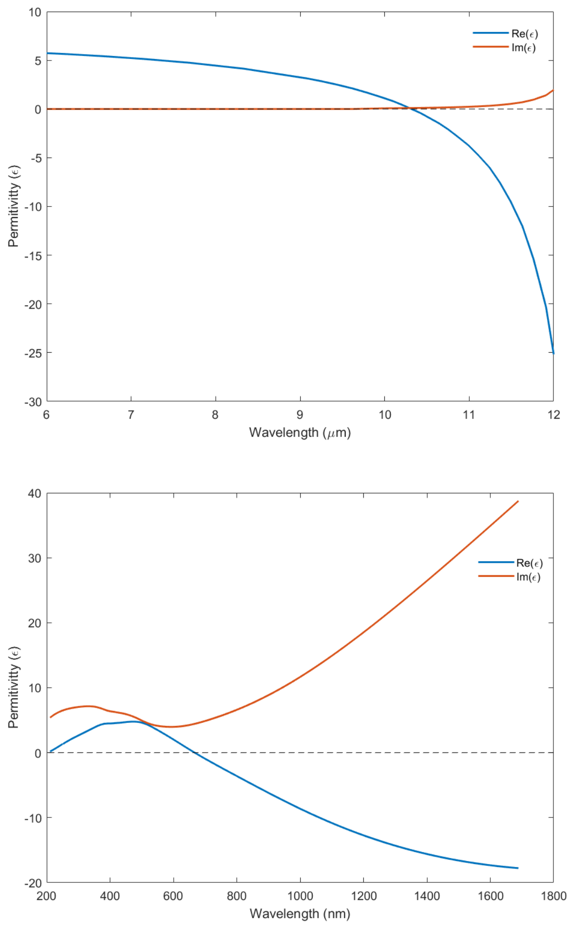


Figure 3: **Permittivity of SiC and TiN**: Real (blue) and imaginary (red) curves of the permittivities of SiC (top) and TiN (bottom).

## Intensity profile

In Fig.4 we present a profile of the normalized electric field in the network as a function of the horizontal position, showing that inside the other cavities the field is almost as high as in the one containing the source, represented by the blue arrow.

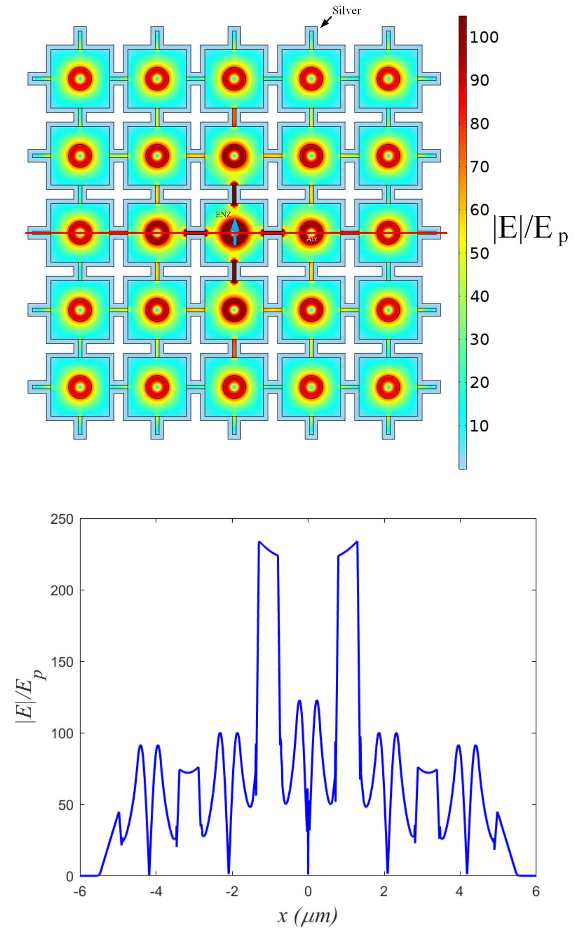


Figure 4: **Electric field profile of the 2D ENZ Quantum Network**. Normalized electric field profile,emitted by a point source placed inside the middle cavity, as a function of the horizontal cut line (red line).

## Curve fitting

Data analysis for bigger networks was carried while maintaining constant the dimensions of all the blocks of the network. We have found the following relation between the magnetic field of the central cavity and the number of cavities

$$\frac{|H|}{|H_{p3x3}|} = 4.63e^{-0.59n} + 0.20, \tag{15}$$

where $\frac{|H|}{|H_{p3x3}|}$ is the normalized magnetic field with respect to the point source located at the central cavity with lowest unitary cell, given by 3x3 cavities, and $n$ is the total number of cavities in one direction of the network. Fig. 5 depicts the plot of the data for the magnetic field and the blue curve corresponds the fitting for the above equation.

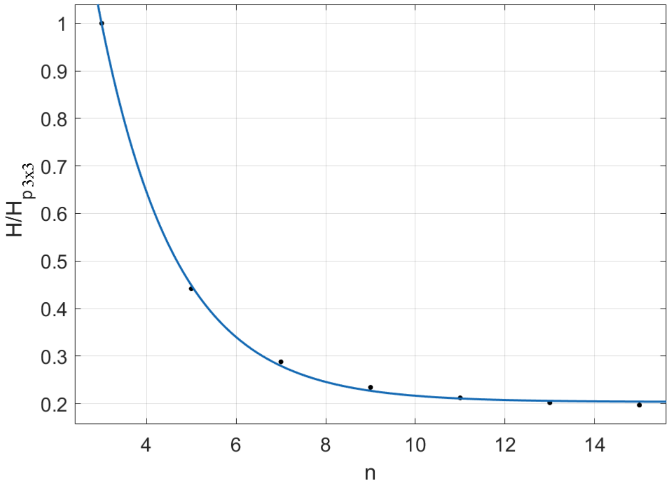


Figure 5: **Fitting analysis**: a) Plot of the normalized magnetic field, at the central cavity, as a function of the number of cavities in one direction of the network. The blue curve represents the fitting.

For the analysis of how the field profile decays with the distance from the middle cavity we have collected the maximum values of the normalized magnetic field in each cavity of networks consisting of 25, 49, 91, 121, 169 and 225 cavities. Since the profile obtained is symmetric, it was enough to analyze only positive values of the

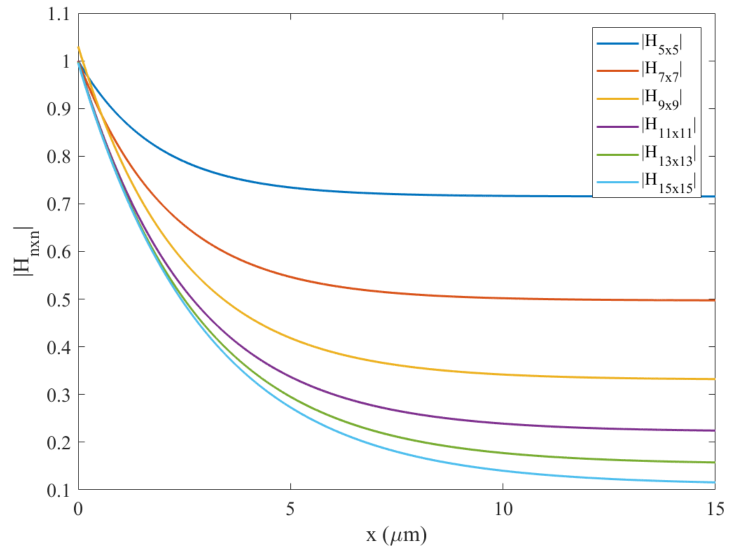


Figure 6: **General expression**: Plot of the relation between the normalized magnetic field for networks of different sizes and the position of each cavity located at the right side of the central cavity, where the blue curve represents the fitting.

position of the cavities, this is, cavities placed at the right side from the central one. The decay of the magnetic field as a function of distance $x$ from the source is plotted in Fig.6 for different cavity sizes, following equation

$$|H_{nxn}| = \frac{4.633}{n} + \left(\frac{-4.608}{n} + 1.19\right) e^{-(1.533/n+0.2409)x} - 0.1934. \tag{16}$$

In this context we analyzed bigger networks containing much more nodes in order to see their limitations. We chose to look on the peak of the magnetic field, due to its monopole distribution inside the cavity. The drop of the peak value served as an unambiguous parameter to quantify intensity distribution within the network (see Fig. 7). As long as we maintain the same dimensions of all blocks of the network as in the case in Fig. 4 effectively supporting the ENZ regime, we find, for this particular network geometry, the following

relation between the magnetic field and the number of cavities

$$|H_{nxn}| = \frac{4.633}{n} + \left(\frac{-4.608}{n} + 1.19\right) e^{-(1.533/n+0.2409)x} - 0.1934, \tag{17}$$

where $|H_{nxn}|$ is the normalized magnetic field with respect to the point source located at the central cavity, $n$ is the total number of cavities in one direction of the QN and $x$ is the distance from the center. Through this result we can estimate the drop of the intensity with the size of this ENZ network without spending computational time on extended simulations. The decay of the magnetic field as a function of the position of the cavities, on the $x$ axis in the ENZ QN, from the source is represented by the red curve in Fig.7(b) and is fitted by equation (17), for $n = 15$.

(a)

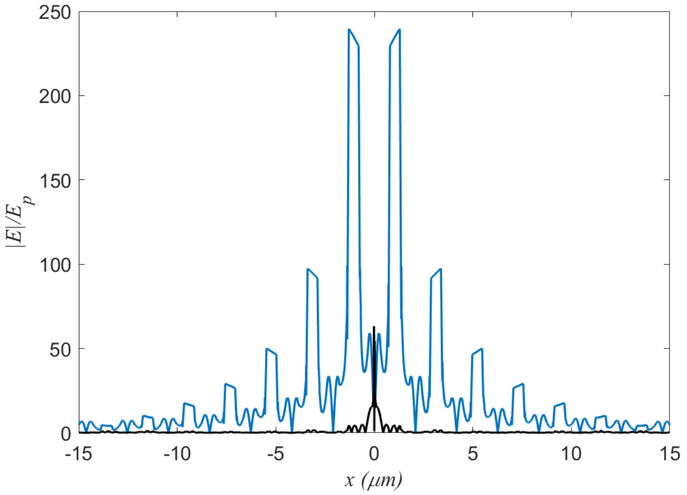


(b)

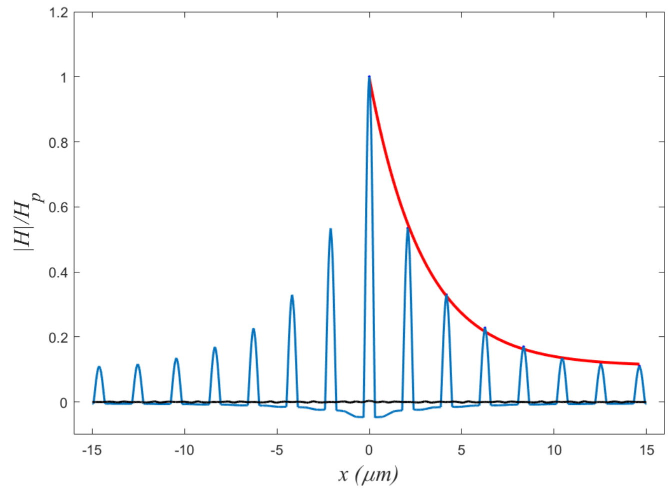


Figure 7: **Comparison between air and ENZ waveguides**.(a) Normalized electric field profile, as a function of the distance from the middle cavity, for an ENZ network (blue) and air network (black), both with the same number of cavities. (b) Normalized magnetic field profile of the same system for an ENZ network (blue) and air network (black) and fitting curve of the maximum values of the magnetic field (red).

## Bigger networks

Simulation results for a 2D ENZ quantum network with 15x15 cavities. Fig. 8 a) shows the normalized electric field emitted by a dipole placed at the center of the middle cavity. The phase distribution of this system is depicted in Fig. 8 b), where the blue arrow represents the position of the dipole.

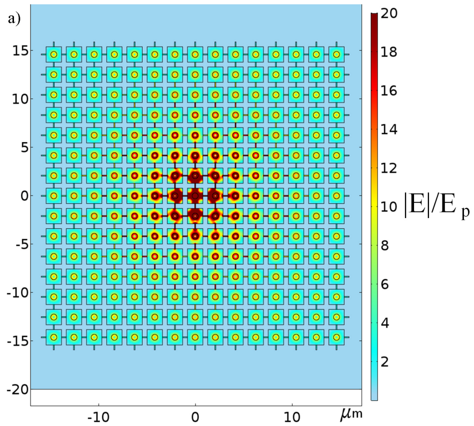


Figure 8: **Electric field profile of the 2D ENZ Quantum Network**. a) Normalized electric field profile, emitted by a point source placed inside the middle cavity. b) Phase distribution.

## References

[1] Silveirinha, M. G. and Engheta, N., Physical Review B, **76**, 245109 (2007).

[2] Comsol multiphysics, R v. 5.2. www.comsol.com. comsol ab, stockholm, sweden. URL www.comsol.com.

[3] I. Liberal and N. Engheta, Science Advances, **2**, e1600987 (2016).

[4] H. Raether, Excitation of Plasmons and Interband Transitions by Electrons, Springer Tracts in Modern Physics (Springer Berlin Heidelberg, 2006).

[5] B. Saleh and M. Teich, Fundamentals of Photonics, Wiley Series in Pure and Applied Optics (Wiley, 2013).

[6] E. Shkondin, T. Repän, O. Takayama, and A. Lavrinenko, Optical Materials Express, **7**, 4171 (2017).

[7] Palik, E. Handbook of Optical Constants of Solids. v. 2 (Elsevier Science, 2012).
